# Outcomes of mechanical and bioprosthetic surgical valve replacements in egypt: evidence from a narrative review and experts’ survey

**DOI:** 10.1186/s13019-026-04400-6

**Published:** 2026-06-10

**Authors:** Mohamed El Fiky, Ahmed Ahmed, Emad Sarawy, Mohamed El Ghanam, Mohamed Sanad, Tamer Mansour, Baher Elezbawy, Ahmad Fasseeh, Sara Essam, Ahmad Mowafy, Sherif Abaza, Adham Salem, Elhusseiny Gamil

**Affiliations:** 1 Department of Cardiothoracic surgery, Faculty of Medicine, Ain Shams University, Cairo, Egypt; 2 Department of Cardiology, Nasser Health Institute, Cairo, Egypt; 3 Department of Cardiac Surgery, National Heart Institute, Giza, Egypt; 4 Cardiothoracic and vascular surgery Academy, Cairo, Egypt; 5 Cardiothoracic and Vascular Surgery Center (CVSC), Mansoura University Hospitals, Mansoura, Egypt; 6 Military Medical Academy, Cairo, Egypt; 7 Maadi Armed Forces Medical Complex, Cairo, Egypt; 8 Doctoral School of Pharmaceutical Sciences, Semmelweis University, Budapest, Hungary; 9 Syreon Middle East, Alexandria, Egypt; 10 Faculty of Pharmacy, Alexandria University, Alexandria, Egypt; 11 Edwards Lifesciences , Dubai, United Arab Emirates; 12 Department of Cardiothoracic surgery, Faculty of Medicine, Al-Azhar University, Cairo, Egypt

**Keywords:** Heart valves, SAVR, SMVR, Egypt, Mechanical valves, Bioprosthetic valves, Tissue valves

## Abstract

**Background:**

Selecting between mechanical and bioprosthetic valves depends on multiple clinical and patient-specific criteria. This study aims to compare mechanical and bioprosthetic valve replacement outcomes in Egypt to support evidence-informed decision-making.

**Methods:**

We conducted a narrative literature review to identify studies reporting outcomes of mechanical and bioprosthetic valve replacements in Egypt. Findings were validated through an expert meeting with cardiothoracic surgeons. To capture real-world practice, an online survey was administered to cardiothoracic surgeons across Egypt. The narrative review and survey results were compared to global data to formulate recommendations on optimizing valve selection in Egypt. We extracted data from 41 eligible studies identified from the literature.

**Results:**

Findings showed that unlike global data, reoperation rate in Egypt is lower with bioprosthetic valves compared to mechanical valves. Mechanical valves are associated with higher bleeding rates globally and locally. Survival and stroke incidence do not differ significantly between mechanical and bioprosthetic valves. The expert survey was responded to by 128 cardiothoracic surgeons. Experts predominantly used mechanical valves, highlighted age, and anticoagulation eligibility as the main factors influencing valve choice, and showed that longer clinical experience is associated with more tendency to use bioprosthetic valves.

**Conclusions:**

Valve type selection in Egypt should be individualized and made on a case-by-case basis due to the multiple factors influencing choice and outcomes. To support evidence-based decision-making, more local studies should be conducted, especially for bioprosthetic valves. These factors should be considered in selection to choose the most suitable valve for each patient.

**Supplementary Information:**

The online version contains supplementary material available at 10.1186/s13019-026-04400-6.

## Highlights

Selection between mechanical or bioprosthetic valve is a complex process and decision should be individualized.

Global data about valve surgery outcomes can be used, but more local data are required to better inform evidence-informed decision-making.

Reoperation rates are substantially different between Egypt and global data.

**Supplementary Information:**

The online version contains supplementary material available at 10.1186/s13019-026-04400-6.

## Background

Surgical valves replacement involves two main types of prosthetic valves: mechanical and bioprosthetic (tissue) valves. The choice depends on patient characteristics, availability, cost, and surgeon preference [1]. Mechanical valves, made from durable materials like carbon or titanium, can last up to 40 years but require lifelong anticoagulation to prevent clot formation. Bioprosthetic valves, typically derived from animals like horses, pigs or cows, last about 10–20 years, then reoperation for valve replacement becomes necessary. Their main advantage is eliminating the need for lifelong anticoagulation [2, 3].

Therefore, the decision to choose the most suitable valve is patient-specific, and depends on several factors, of which age is the most influential. Mechanical valves are generally favored in younger patients to reduce the need for reoperation. Bioprosthetic valves are generally preferred in older patients, to reduce the need of anticoagulation. However, several additional factors contribute to the decision, of which the most important is tolerability to anticoagulation [2, 3].

Replacing a heart valve is considered a major procedure, carrying severe risks, impacting quality of life, and incurring high costs [4]. Healthcare decisionmakers aim to avoid reoperations whenever possible through choosing the optimal valve for each patient. Because each valve type is associated with advantages and disadvantages, a personalized decision is necessary to choose the optimal valve type [5].

Heart valve surgeries are well established in Egypt, supported by experienced surgeons and advanced infrastructure such as the National Heart Institute and Magdi Yacoub foundation, which together perform more than 4,000 open heart surgeries annually, in addition to other public and private institutions [6, 7]. However, in Egypt, the choice of valve type is still inadequately supported by local evidence on durability, complications, and long-term clinical outcomes. This evidence is necessary for surgeons to make evidence-informed decisions. Although several individual studies reported outcomes for mechanical and bioprosthetic valves, no comprehensive studies have compiled these findings to provide a clear and concise overview on the long-term outcome for both valve types in Egypt.

Globally, there is sufficient data about heart valves surgery outcomes, such as the recent systematic literature review by Vankayalapati et al., which examines the long-term outcomes of mechanical versus bioprosthetic aortic valve replacement [8], the survival and reoperation rates analysis by Feirer et al.^9^, and the long-term outcomes analysis by Chikwe et al. ^9, 10^. However, it remains uncertain if global data are fully applicable to Egypt, as outcomes may differ due to unique population characteristics, behavior or social factors.

In the absence of a publicly accessible patient registry for heart valve patients in Egypt, data primarily come from locally conducted studies. However, these studies may not reflect a full capture of real-world clinical experience. Therefore, there is a need for evidence that combines data from the literature with insights from clinical experts reflecting the actual outcomes observed.

This study aims to summarize the long-term outcomes of surgical mechanical and bioprosthetic heart valve replacements in Egypt, gather expert opinions on these outcomes, and integrate both literature and real-world experiences to provide a comprehensive understanding of valve replacement surgery outcomes in Egypt. The findings aim to support decision makers to choose the most optimal valve for each patient.

The primary objective was to compare reoperation rates of mechanical versus bioprosthetic valves in surgical aortic valve replacement (SAVR) and surgical mitral valve replacement (SMVR) in Egypt, and to benchmark these data against global data. The secondary objectives were to evaluate the risk of complications, such as mortality, bleeding and thromboembolic events, among both valve types, and to compare Egyptian-specific outcomes against global data. Finally, we aimed to determine whether global clinical data are applicable to the Egyptian settings, or if unique population characteristics result in different outcomes.

## Methods

We conducted a narrative review to summarize studies reporting surgical valve outcomes conducted in Egypt, and compared these findings to global data. Following this, we held a validation meeting with a sample of experienced cardiothoracic surgeons to present and discuss the review findings. To incorporate real-world clinical insights, we created an online survey for cardiothoracic surgeons practicing in Egypt to further validate and complement the literature findings. Finally, we integrated and analyzed the results from all sources to draw comprehensive conclusions about surgical heart valve surgery outcomes in Egypt, and to provide recommendations for optimizing heart valves selection in Egypt.

The whole research was conducted through the collaboration of three key parties. A research team, which was responsible for the study design, narrative review, data analysis, survey development, and interpretation of the results. A panel of experienced cardiothoracic surgeons, who served as expert advisors, participating in the study design, validation of each stage, and refinement key steps to ensure the robustness and relevance of the outcomes. Finally, a group of cardiothoracic surgeons participated anonymously by completing the survey, enriching the study outcomes with real-world experience.

### Narrative review

Based on a preliminary literature search and discussions with the experienced cardiothoracic surgeons, the research team identified several key outcomes for evaluation: reoperations rates, complications, and compliance to anticoagulation. These outcomes were the main focus of the narrative review.

The review aimed to address three main research questions. The first was to determine the reoperation rates of mechanical versus bioprosthetic valves in SAVR and SMVR in Egypt. The second question focused on identifying the differences in the risk of mortality, bleeding and thromboembolic complications between mechanical and bioprosthetic valve recipients in Egypt. The third was to assess the compliance rate to anticoagulation among mechanical valve replacement recipients in Egypt.

To ensure comprehensiveness, we searched all potential sources that might include outcomes data on SMVR and SAVR surgeries in Egypt published from January 2010 onward. The 2010 cutoff was selected to reflect the advancements in valve durability and changes in anticoagulation practices recently. The search covered peer reviewed literature (PubMed, Embase, Cochrane reviews, Google scholar, The Egyptian Society of Cardio-Thoracic Surgery’s journal), clinical trial databases (Clinical trials.gov, EU Clinical Trials Register), and grey literature sources (Egyptian Knowledge Bank, Egyptian Universities Libraries Consortium, Google Search).

For the literature search we used a combination of keywords including “mechanical valve”, “bioprosthetic valve”, “heart valve replacement”, “reoperation”, “bleeding”, “thromboembolism”, “Egypt”, and “compliance” or “adherence.” We included both observational studies and randomized controlled trials (RCTs) to ensure a comprehensive assessment of outcomes.

We included studies involving adult populations who underwent mechanical or bioprosthetic SAVR or SMVR in Egypt. A summary of the inclusion criteria is presented in the PICOS (Patients, Intervention, Comparator, Outcomes, Study design) table in Additional file 1.

We screened the identified references by titles and abstracts, followed by full-text review, applying the following exclusion criteria: (1) Published before 2010; (2) studies not in English or Arabic; (3) animal or invitro studies; (4) duplicate or reporting overlapping data; (5) non-adult population; (6) irrelevant valve type (pulmonary/tricuspid or mixed with other types); (7) irrelevant implant type (not mechanical or tissue valves); (8) Irrelevant study design; (9) no relevant outcomes (reoperation, mortality or survival, bleeding, thromboembolic events, compliance to anticoagulation of valve replacement recipients); (10) other reasons (e.g. studies with specific confounders rendering outcomes non-comparable).

For included studies, a data extraction sheet was designed to extract data on both single arm outcomes (e.g. 20% reoperation rate/reoperation after an average of 3 years), and comparative outcomes (e.g. odds ratios/relative risks). Comparative outcomes were restricted to direct comparisons between mechanical and bioprosthetic valves. The components of the data extraction sheet are provided in Additional file 2.

We extracted five primary outcome categories: reoperation rates, survival, bleeding, stroke, and compliance to anticoagulation. These outcomes were assessed across several studies conducted in Egypt and were compared between patients receiving mechanical valves and those receiving tissue valves. Additionally, these outcomes were compared to findings from a global systematic literature review to compare outcomes between local and international data [8].

Based on the review findings, a validation meeting was conducted with the research team members and a sample of five experienced cardiac surgeons in Egypt. During the meeting, the research team presented the literature review results and engaged with the experts to validate the outcomes and identify the knowledge gaps. These gaps informed the development of an expert questionnaire, which was used to complement the literature findings by capturing real-world expert insights. The questionnaire was subsequently reviewed and validated during the meeting to ensure its clarity and relevance.

### Experts survey

The expert survey included 25 questions, grouped into five sections: demographics, valve selection criteria and preferences, clinical outcomes and complications, expert insights, and Egyptian versus global context. The full survey is available in Additional file 3.

The survey was disseminated through Google forms and targeted exclusively at cardiothoracic surgeons. The inclusion criterion was to be an officially registered cardiothoracic surgeon in Egypt. No additional restrictions were applied. To ensure the eligibility of respondents, the questionnaire was disseminated through a single channel - an existing online group limited to cardiothoracic surgeons in Egypt who are members of The Egyptian Society of Cardiothoracic Surgery. This society is the largest professional body in Egypt, comprising more than 700 members [11]. The society’s members represent the majority of practicing cardiothoracic surgeons in Egypt, and are affiliated with a wide range of public and private institutions across different geographical regions of the country.

The survey responses were analyzed using descriptive statistics. In addition, thematic analysis was employed to show insightful patterns of the results. To minimize potential bias, experts who responded to the survey were not exposed to the literature review findings prior to responding.

### Drawing conclusions and formulating recommendations

Based on the narrative review and experts survey findings, the research team combined the outcomes, synthesized the results, and formulated them into draft conclusions. These were then formulated into final conclusions and recommendations in collaboration with the experienced cardiothoracic surgeons in an online meeting that aimed to synthesize the evidence from all research findings.

### Data processing

Heterogenous data were cleaned and standardized to enable inclusion in the analysis and data were harmonized to achieve consistency.

Several data points were reported for specific patient subgroups, such as pregnant women, children, and patients with specific characteristics. To minimize potential bias, we excluded these from analysis and used only the main population values. Similarly, highly specific outcomes such as specific types and locations of bleeding (e.g. epistaxis) and certain thromboembolic events (e.g. non-cerebral embolic events) were also excluded. Only primary outcome data were included in analysis.

Studies reported outcomes using varying terminology; therefore, standardized definitions were applied. Reoperation was defined as any subsequent valve replacement after the primary surgery. Survival was defined as all-cause survival or calculated as 1 minus mortality. Bleeding included overall or major bleeding, excluding minor or cause-specific events. Stroke was defined as valve-related ischemic stroke. Compliance with anticoagulation was defined as receiving therapy and attending ≥ 80% of scheduled doses or monitoring visits.

Studies reported outcomes at heterogeneous timepoints. To ensure comparability, data were grouped into predefined intervals: intraoperative, 0–14 days, 15–30 days, 1 month, 3 months, 6 months, 1 to < 2 years, 2-<3 years, 3 years, 5 years, and 10 years. Absence of data at a given timepoint indicates that no studies reported that outcome at that interval.

For the survey outcomes, categorical variables were summarized as frequencies and percentages. Associations between respondents’ characteristics (affiliation, position, and years of experience) and valve type preference were assessed using the chi-square test or Fisher’s exact test, as appropriate. A p-value of < 0.05 was considered statistically significant.

## Results

### Narrative review

**Fig. 1:**
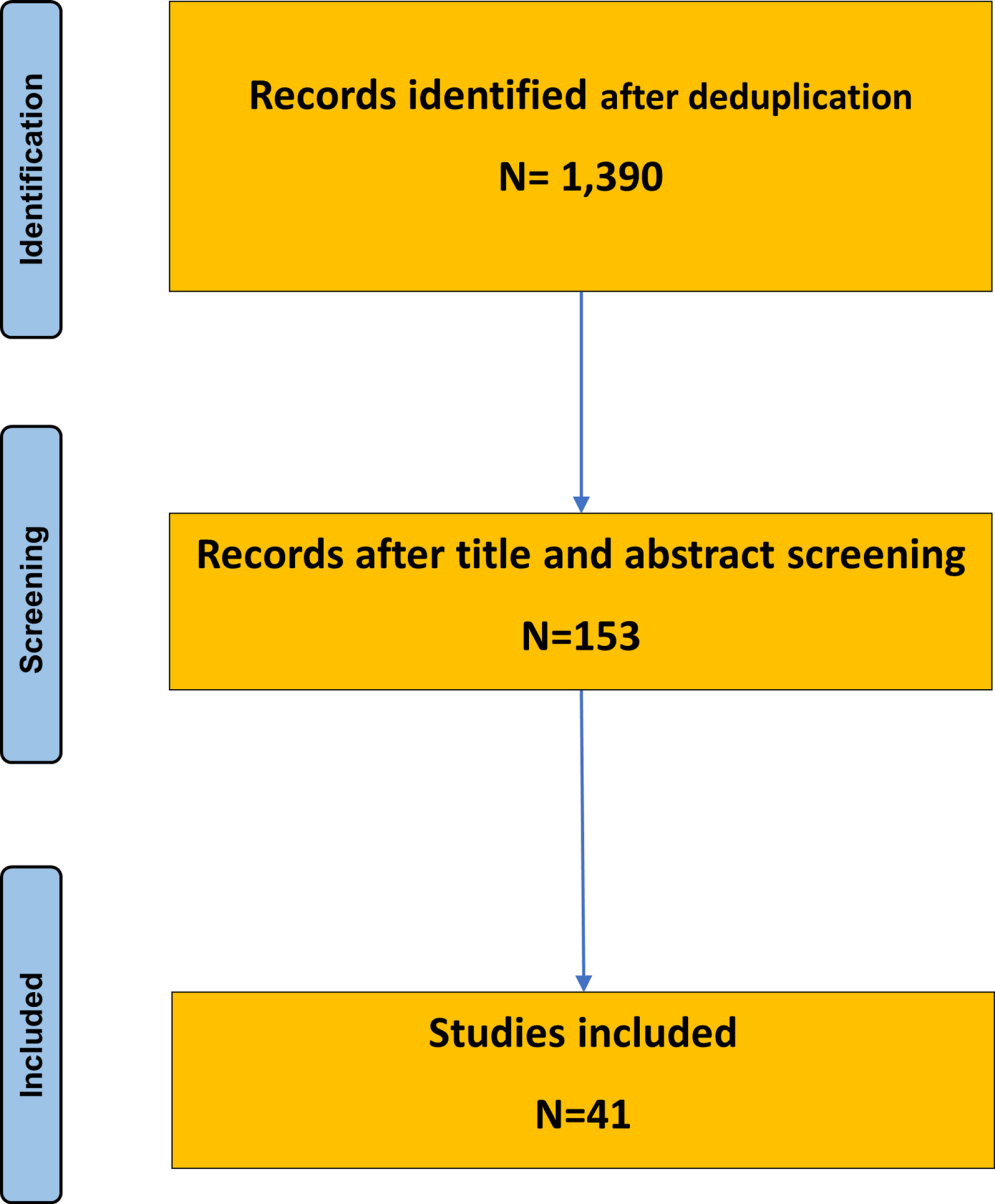
Study inclusion flow diagram

We identified 1,390 records through the search process, including 365 studies from peer-reviewed sources, and 1,025 from grey literature. After title and abstract screening, 153 studies were deemed relevant for full text review. Of these, 41 studies met the inclusion criteria and were included in the final quantitative data analysis. Figure 1 below illustrates the study selection process.

## Single arm outcomes of mechanical and bioprosthetic valves in Egypt

### Reoperation

Few studies reported data on reoperation, especially for bioprosthetic valves. For mechanical valves, the reported 3-year reoperation rates were low, with no reoperations (0.0%) observed among patients receiving a combination of aspirin and anticoagulants, and 3.8% among patients receiving either oral anticoagulants or vitamin K antagonists [12].

Three studies reported 5-year reoperation rates following mechanical mitral valve replacements with rates ranging from 4.2% to 12.5%. Notably, the study by Samy et al. focused on a specific cohort of females in childbearing age [13], while the two other studies [14, 15] included broader populations with rheumatic heart disease. The study by Samy et al. included patients with both types of valves. In this study, the 5-year reoperation rate with bioprosthetic valves was reported at 1.7%, compared to 11.7% with mechanical valves.

At 10 years, Singab et al. [16] reported reoperation outcomes among 355 young females who have undergone mitral mechanical or bioprosthetic valve replacement. In this population, the 10-year reoperation rate was 3.7% for mechanical valves compared to only 0.6% for bioprosthetic valves.

Both data points for reoperation rates among bioprosthetic valves recipients were derived from studies that included young female populations.

Two studies reported the time to reoperation with mechanical valves, while no such data was available for bioprosthetic valves. Lamloum (2015) reported a mean time to reoperation of 5.62 years (*n* = 60) [17], while Mahmoud (2015) found a similar duration of 5.29 years (*n* = 40) [18]. Omar et al. (2020) reported reoperation rates among 104 mechanical mitral valve replacements, with rates increasing from 3.85% in the first month (early reoperation), to 12.5% at 5 years (including 4 early and 9 late reoperations) [14].

Singab et al. (2020) reported lower odds of reoperation in patients with bioprosthetic valves compared to those with mechanical valves (OR = 0.14; 95% CI: 0.0175–1.180), although the difference was not statistically significant [16].

Table 1 below provides a summary of reoperation rates reported across the included studies.

**Table 1:** Reoperation rates on bioprosthetic and mechanical valves

Study	Number of patients	Cohort short description	Type of valve	Reoperation on bioprosthetic	Reopration on mechanical
**3 years**					
Fawzy_2017 [12]	104	Patients using oral anticoagulants + aspirin after operation	Mitral	--	0.0%
Fawzy_2017 [12]	79	Patients using oral anticoagulants only or Vitamin K Antagonists after operation	Mitral	--	3.8%
**5 years**					
Kassem_2024 [15]	96	Patients with rheumatic heart disease	Mitral	--	4.2%
Samy_2019 [13]	Bioprosthetic: 120 Mechanical: 120	Female patients in childbearing period	Mitral	1.7%	11.7%
Omar_2020 [14]	104	Patients with rheumatic heart disease	Mitral	--	12.5%
**10 years**					
Singab_2020 [16]	Bioprosthetic: 174 Mechanical: 181	Female patients in childbearing period	Mitral	0.6%	3.7%

### Survival

Several studies reported survival or mortality data after surgical valve replacement at different timepoints and in various clinical scenarios. Below, we summarize the studies that reported overall mortality or survival of patients after SAVR or SMVR. The results are summarized in Table 2.

Only five studies reported survival data for both patient groups (mechanical valves and bioprosthetic valves). At day 0 postoperatively, Singab et al. (2020) [16] reported 100% survival in the bioprosthetic group (*n* = 174) compared to 99.4% with mechanical group(*n* = 181). At one month, Ali et al. (2021) [19] reported identical survival with both groups at 96.0% (*n* = 25 each). At six months, Hassan et al. (2019) [20] reported survival rates at 94.4% in the bioprosthetic group (*n* = 72) compared to 96.1% for mechanical group (*n* = 128). At 5 years Samy et al. (2019) [13] reported 100% survival with bioprosthetic (*n* = 120) and 99.2% with mechanical (*n* = 120). Finally, Singab et al. (2020) [16] also reported long-term survival at 10 years, showing a shift toward better outcomes with bioprosthetic valves at 96.4% (*n* = 174) compared to mechanical valves at 90.0% (*n* = 181).

**Table 2:** Summary of reported survival outcomes

Study	Number of patients	Cohort short description	Type of valve	Survival on bioprosthetic	Survival on mechanical
**Intraoperative/0 days**					
Saleh_2021 [21]*	97	Patients who received an emergency redo operation for valve thrombosis	Aortic and mitral	--	74.2%
Dokhan_2016 [22]*	40	Redo surgery patients	Mitral	--	87.5%
Gamil_2022 [23]	50	Young and middle-aged adults (15–45 yrs)	Aortic	--	96.0%
Faisal_2024 [24]	30	Adults with infective endocarditis	Mitral	--	96.7%
AbdElGhaffar_2014 [25]	248	Adults	Mitral	--	98.8%
Singab_2020 [16]	Bioprosthetic: 174 Mechanical: 181	Female patients in childbearing period	Mitral	100.0%	99.4%
Kasab_2020 [26]	39	All valve replacement patients	Aortic and mitral	--	100.0%
**0–14 days**					
Hassan_2021 [27]*	37	Patients who received a redo operation	Mitral	--	91.89%
Gamil_2022 [23]	50	Young and middle-aged adults (15–45 yrs)	Aortic	--	98.00%
**15–30 days**					
Mahmoud_2015 [18]*	40	Patients who received a redo operation for valve thrombosis or malfunction	Mitral	--	82.5%
**1 month**					
Saleh_2021 [21]*	97	Patients who received an emergency redo operation for valve thrombosis	Aortic and mitral	--	67.0%
Elghanam_2023 [28]*	40	Redo valve replacement for malfunction	Mitral	--	82.5%
Hassan_2021 [27]*	37	Patients who received a redo operation	Mitral	--	89.2%
Rizk_2018 [29]	30	Patients with ascending aorta aneurysm	Aortic	--	93.3%
El Midany _2019 [30]	715	All valve replacement patients	Mitral	--	94.8%
Ali_2021 [19]	Bioprosthetic: 25 Mechanical: 25	Ischemic patients undergoing chordal‑sparing valve replacement	Mitral	96.0%	96.0%
Kasab_2020 [26]	39	All valve replacement patients	Aortic and mitral	--	97.4%
**3 months**					
El Barbary_2023 [31]	10	Valve replacement patients	Aortic	--	90.0%
AbdElGhaffar_2014 [25]	248	Adults	Mitral	--	90.3%
Faisal_2024 [24]	30	Adults with infective endocarditis	Mitral	--	96.7%
**6 months**					
Dokhan_2016 [22]*	40	Redo surgery patients	Mitral	--	80.0%
Shalaby_2020 [32]	20	Valve replacement patients	Mitral	--	90.0%
Nour_2022 [33]	20	Adults > 65 years	Aortic	--	95.0%
Hassan_2019 [20]	Bioprosthetic: 72 Mechanical: 128	Middle aged adults with rheumatic disease	Aortic	94.4%	96.1%
Sarawy_2020 [34]	25	minimally invasive valve replacement (Mini sternotomy)	Aortic	--	100.0%
Sarawy_2020 [34]	25	Standard full sternotomy	Aortic	--	100.0%
**1 to < 2 years**					
Kasab_2020 [26]	39	All valve replacement patients	Aortic and mitral	--	94.87%
**2 to < 3 years**					
El Midany _2019 [30]	715	All valve replacement patients	Mitral	--	91.00%
**5 years**					
Omar_2020 [14]	104	Patients with rheumatic heart disease	Mitral	--	90.4%
Kassem_2024 [15]	96	Patients with rheumatic heart disease	Mitral	--	93.2%
Samy_2019 [13]	Bioprosthetic: 120 Mechanical: 120	Female patients in childbearing period	Mitral	100.0%	99.2%
**10 years**					
Singab_2020 [16]	Bioprosthetic: 174 Mechanical: 181	Female patients in childbearing period	Mitral	96.4%	90.00%

*Patients with redo valve surgery

### Bleeding

For mechanical valves, short-term bleeding (0–14 days) ranged from 1.64% to 52.00%. Long-term bleeding, assessed from 3 months up to 5 years, ranged from 1.67% to 20.00% at various time points, as shown in Table 3.

Two studies, Samy et al. (2019) [13] and Mourad et al. (2016) [36], reported comparative data on both mechanical and bioprosthetic valves. In Samy et al., at 5 years timepoint, 5.83% of the patients had bleeding events with mechanical valves, while no bleeding events were observed with bioprosthetic valves (0.0%) during the same period.

Mourad et al. (2016) reported more specific bleeding outcomes. No reoperation for bleeding and no anticoagulant-related bleeding events occurred in patients who received bioprosthetic valves at 1-year post-surgery, while 3.30% of patients who received mechanical valves had reoperation for bleeding, and 20.0% had anticoagulant-related bleeding events during the same period (*n* = 30 in each group).

**Table 3:** Bleeding frequency with mechanical versus bioprosthetic valves

Study	Number of patients	Cohort short description	Type of valve	Bleeding on bioprosthetic	Bleeding on mechanical
**0–14 days**					
Youssef_2013 [37]	61	Valve replacement for isolated or mixed aortic valve lesions	Aortic	--	1.64%
Gamil_2022 [23]	50	Young and middle-aged adults (15–45 yrs)	Aortic	--	6.00%
Lamloum_2015 [17]*	60	Redo valve replacement for malfunction	Mitral	--	16.67%
Sabry_2022 [35]	25	Patients on warfarin 3 mg	Mitral	--	32.00%
Sabry_2022 [35]	25	Patients on warfarin 5 mg	Mitral	--	52.00%
**3 months**					
Ahmed_2017 [38]	60	25–65 adults for elective valve replacement	Mitral	--	1.67%
Faisal_2024 [24]	30	Adults with infective endocarditis	Mitral	--	16.67%
El Barbary_2023 [31]	10	Valve replacement patients	Aortic	--	20.00%
**6 months**					
Nour_2022 [33]	20	> 65 year adults	Aortic	--	20.00%
**3 years**					
Mostafa_2018 [39]	360	Valve replacement patients	Mitral	--	3.33%
**5 years**					
Samy_2019 [13]	Bioprosthetic: 120 Mechanical: 120	Female patients in childbearing period	Mitral	0.00%	5.83%
Kassem_2024 [15]	96	Patients with rheumatic heart disease	Mitral	--	7.30%

*Patients with redo valve surgery

### Stroke

In patients who received mechanical valves, reported stroke proportions ranged from 0.00% up to 5.00%. Two studies with patients on redo valve surgery showed higher proportions of stroke at 7.50% and 8.11%, ^18, 28^ as shown in Table 4.

For patients who received bioprosthetic valves, only one study -Singab et al.- reported stroke outcomes, with 1.10% of patients had stroke among 174 patients. In the same study, stroke frequency was reported at 4.40% for patients who received mechanical valves (*n* = 181) [16].

### Compliance to anticoagulation

Among patients receiving mechanical valves, data on compliance with anticoagulation therapy was limited in the included studies. Only two studies -Saleh et al., 2021 ^21^ and Singab et al., 2020 ^16^- reported specific information about compliance to anticoagulation therapy.

In Saleh et al., among 97 patients with mechanical valve replacement, 61% were inadequately anticoagulated at admission. Additionally, the study reported that 52.5% of patients had regular follow-up of their anticoagulation status after valve replacement, while the others either missed their visits, or had irregular monitoring.

Singab et al., reported a high compliance rate of 81%. In this cohort, Singab et al. mentioned that oral anticoagulants were consistently available and accessible to the patients.

**Table 4:** Stroke frequency with mechanical versus bioprosthetic valves

Study	Number of patients	Cohort short description	Type of valve	Stroke on bioprosthetic	Stroke on mechanical
**0–14 days**					
Kasab_2020 [26]	39	Valve replacement patients	Aortic and mitral	--	2.56%
**1 month**					
Singab_2020 [16]	Bioprosthetic: 174 Mechanical: 181	Female patients in childbearing period	Mitral	1.10%	4.40%
Elghanam_2023 [28]*	40	Redo valve replacement for malfunction	Mitral	--	7.50%
Mahmoud_2015 [18]*	37	Patients who received a redo operation for valve thrombosis or malfunction	Mitral	--	8.11%
**3 months**					
El Barbary_2023 [31]	10	Valve replacement patients	Aortic	--	0.00%
AbdElGhaffar_2014 [25]	248	Adults	Mitral	--	3.20%
**6 months**					
Shalaby_2020 [32]	20	Valve replacement patients	Mitral	--	5.00%
**3 years**					
Fawzy_2017 [12]	104	Patients using oral anticoagulants + aspirin after operation	Mitral	--	0.00%
Fawzy_2017 [12]	79	Patients using oral anticoagulants only or Vitamin K Antagonists after operation	Mitral	--	3.80%
**5 years**					
Gamil_2022 [23]	50	Young and middle-aged adults (15–45 yrs)	Aortic	--	2.00%

*Patients with redo valve surgery

### Comparative studies identified form the narrative review

Only five studies directly compared relevant outcomes for bioprosthetic and mechanical valves as presented in Table 5. The compared populations were heterogenous, limiting the ability to perform direct comparisons of the reported values.

**Table 5:** Studies reporting outcomes for both mechanical and bioprosthetic valve patients

Author_year	Mechanical valves cohort)*n*(	Bioprosthetic valves cohort)*n*(	Study population	Main comparative outcomes reported
Samy_2019 [13]	120	120	Female patients in childbearing period	Reoperation, mortality, bleeding, embolism, valve thrombosis, valve degeneration
Singab_2020 [16]	181	174	Female patients in childbearing period	Reoperation, mortality, stroke, re-exploration
Ali_2021 [19]	25	25	Ischemic mitral patients	Mortality, re-exploration, myocardial infarction
Hassan_2019 [20]	128	72	Middle aged adults with rheumatic disease	Mortality
Mourad_2016 [36]	30	30	Patients with rheumatic mitral valve disease	Reoperation for bleeding

Samy et al. (2019) compared outcomes in 240 females in childbearing age and reported a reoperation frequency of 11.67% with mechanical valves versus 1.67% in the bioprosthetic valves cohort. Five-year mortality was slightly higher at 0.83%, compared to 0.00% with bioprosthetic valves. Bleeding, embolism, and valve thrombosis were only present in the mechanical valves cohort with 5.82%, 4.17%, 5.83% respectively, while none of these complications were observed in the bioprosthetic valves cohort. In contrast, valve degeneration was observed only with bioprosthetic valves at 1.67%.

Singab et al. (2020) reported that reoperation frequency was 3.70% in the mechanical valves cohort compared to 0.60% in the bioprosthetic cohort. At 10 years, mortality was higher with mechanical valves (10.00%) than with bioprosthetic valve recipients (3.60%). Stroke occurred in 4.40% of the patients who received mechanical valves versus 1.10% with bioprosthetic valves. Additionally, re-exploration for bleeding was required in 18% of mechanical valve recipients, compared to 4.00% in the bioprosthetic cohort.

Ali et al. (2021) reported comparable 30-day mortality rates between mechanical and bioprosthetic valve recipients (4.00% each). However, re-exploration was more frequent with mechanical valves (8.00% versus 4.00% for bioprosthetic valves). Similarly, myocardial infarction frequency was also higher in mechanical valve recipients versus bioprosthetic valve recipients (8.00% versus 4.00%).

Mourad et al. (2016) reported the 1-year reoperation for bleeding of 3.30% in the mechanical valves cohort versus 0.00% in the bioprosthetic valves cohort. In contrast, Hassan et al. (2019) observed a higher 6-month mortality with bioprosthetic valves (5.56%) compared to mechanical valves (3.91%).

Of those five studies, only Singab et al. (2020) reported odds ratio values comparing bioprosthetic and mechanical valves in a cohort of young women (bioprosthetic: *n* = 174; mechanical: *n* = 181). Statistically significant lower odds ratios were observed in long-term mortality and re-exploration for bleeding in patients receiving bioprosthetic valves (0.21 and 0.32 respectively). Bioprosthetic valves were also associated with lower reoperation rate, stroke, and short-term mortality, compared to mechanical valves, but those effects were non statistically significant. Results are summarized in Additional file 4.

### Comparison of the outcomes in Egypt to global data

Comparison of global and local findings suggests a divergence in reoperation patterns. Among local studies, only two comparative analyses of mitral valves were identified, both involving females of childbearing age. These reported lower reoperation rates for bioprosthetic versus mechanical valves, with rates of 1.7% versus 11.7% at 5 years in Samy et al.^13^ and 0.6% versus 3.7% at 10 years in Singab et al.^16^ In contrast, global evidence in mitral valve patients from Feirer et al.^9^ and Chikwe et al.^40^ consistently demonstrated lower reoperation rates with mechanical valves across all assessed time points. While differences in study populations limit direct comparability, the available evidence may indicate that Egyptian outcomes do not fully align with the global or Western pattern.

In terms of survival, global data showed a no significant difference among mechanical versus bioprosthetic valves, similar to the few local Egyptian studies which compared survival among mechanical versus bioprosthetic patients, which also showed no difference among both surgeries’ survival. Similarly, in stroke, both local and global data showed no significant difference between bioprosthetic and mechanical valves.

For bleeding events, local and global data were aligned consistently showing lower bleeding rates with bioprosthetic valves.

## Experts’ survey

### Respondents’ demographics

A total of 128 cardiothoracic surgeons responded to the survey, all of whom provided complete and valid responses, corresponding to a response rate of approximately 18%. Most respondents were affiliated with university or academic affiliations (63%), and government or public hospitals (34%), while only 3% were primarily affiliated with private healthcare entities. The respondents demonstrated substantial experience in heart valve surgeries: 34% reported having more than 20 years of experience, and 38% had between 11 and 20 years of experience. Most participating experts (65%) were consultants, and 23% were specialists. The respondents’ demographics reflected a highly experienced pool of experts, enhancing the credibility of the survey’s outcomes. A summary of the full survey responses is available in Additional file 5.

### Valve selection criteria

Concerning valve selection criteria, surgeons showed a strong predominance for mechanical valves, with 89.1% reporting they use mostly mechanical valves and only 2.4% using bioprosthetic valves more, while 8.6% reported not strong preference using both equally. Over the past 5 years, 51% of surgeons reported no change in their use of bioprosthetic valves, while 38.3% reported increased utilization of these valves, and 10.9% reported reduced utilization.

We analyzed the relationship between surgeons’ years of experience and valve type usage to reveal if years of experience have an influence on valve type selection. The results showed that among surgeons with less than 10 years of experience there was no significant difference in valve utilization, while among surgeons with 11–20 years of experience, a non-significant trend was observed towards more bioprosthetic use (*p* = 0.361). A statistically significant increase in bioprosthetic valves utilization was observed among surgeons with more than 20 years of experience (*p* = 0.019) suggesting more experienced surgeons are more likely to use bioprosthetic valves compared to less experienced surgeons.

Respondents emphasized several factors that influence their choice of valve type. The most influential factor by far was patient age, followed by being a female in childbearing age, and eligibility for anticoagulation. In addition, several other factors were reported distinctly for mechanical and for bioprosthetic valves. Table 6 below shows the factors that influence the choice, with an illustration of the proportion of surgeons who selected each factor.

**Table 6:** Factors influencing choice of mechanical and bioprosthetic valves

Factor (*N* = 128)	Mechanical valves		Bioprosthetic valves	
	*n*	%	*n*	%
Patient age (younger vs. older)	119	93.00%	120	93.80%
Being a female in childbearing age	81	63.30%	107	83.60%
Anticoagulation ability/complications	103	80.50%	72	56.30%
Patient comorbidities/life expectancy	65	50.80%	57	44.50%
Warfarin resistance	46	35.90%	55	43.00%
Patient opinion	55	43.00%	49	38.30%
Being a TAVI friendly valve	NA	NA	46	35.90%
Warfarin toxicity	35	27.30%	44	34.40%
Atrial fibrillation	69	53.90%	42	32.80%
Patient preference, lifestyle	54	42.20%	40	31.30%
Availability of a specific type	53	41.40%	39	30.50%
Gender	35	27.30%	37	28.90%
Valve cost or patient affordability	37	28.90%	35	27.30%
Patient’s socioeconomic status	43	33.60%	23	18.00%
Valve size	37	28.90%	22	17.20%
Having an active/sedentary lifestyle	28	14.10%	19	14.80%
Other factors	2	1.60%	1	0.80%

TAVI: Transcatheter Aortic Valve Implantation

Respondents were asked about their preferred valve type across different age groups. While 25% reported no fixed age to switch their choice toward bioprosthetic valves, responses showed a clear age-related trend. As the patient’s age increases, preference for bioprosthetic valves increases, ranging from 2% favoring them in the 20–40 years age group, 5% in 50–60 years age group, 49% in the 60–70 years age group, and reaching 75% in the 70 years and above age group.

### Clinical outcomes and complications

Based on experts’ real-world experiences and perceptions, mechanical valves were estimated to demonstrate longer durability, lasting a mean of 20.8 ± 8.2 years in Egypt, while bioprosthetic valves were estimated to last a mean of 9.0 ± 2.9 years. These estimates reflect aggregated expert opinion rather than measured clinical outcomes.

According to experts’ perceptions, the median compliance rate among patients with mechanical valves in Egypt was estimated at 80% with a range from 40% to 100% (interquartile range: 70%−90%), based on expert perception.

**Fig. 2:**
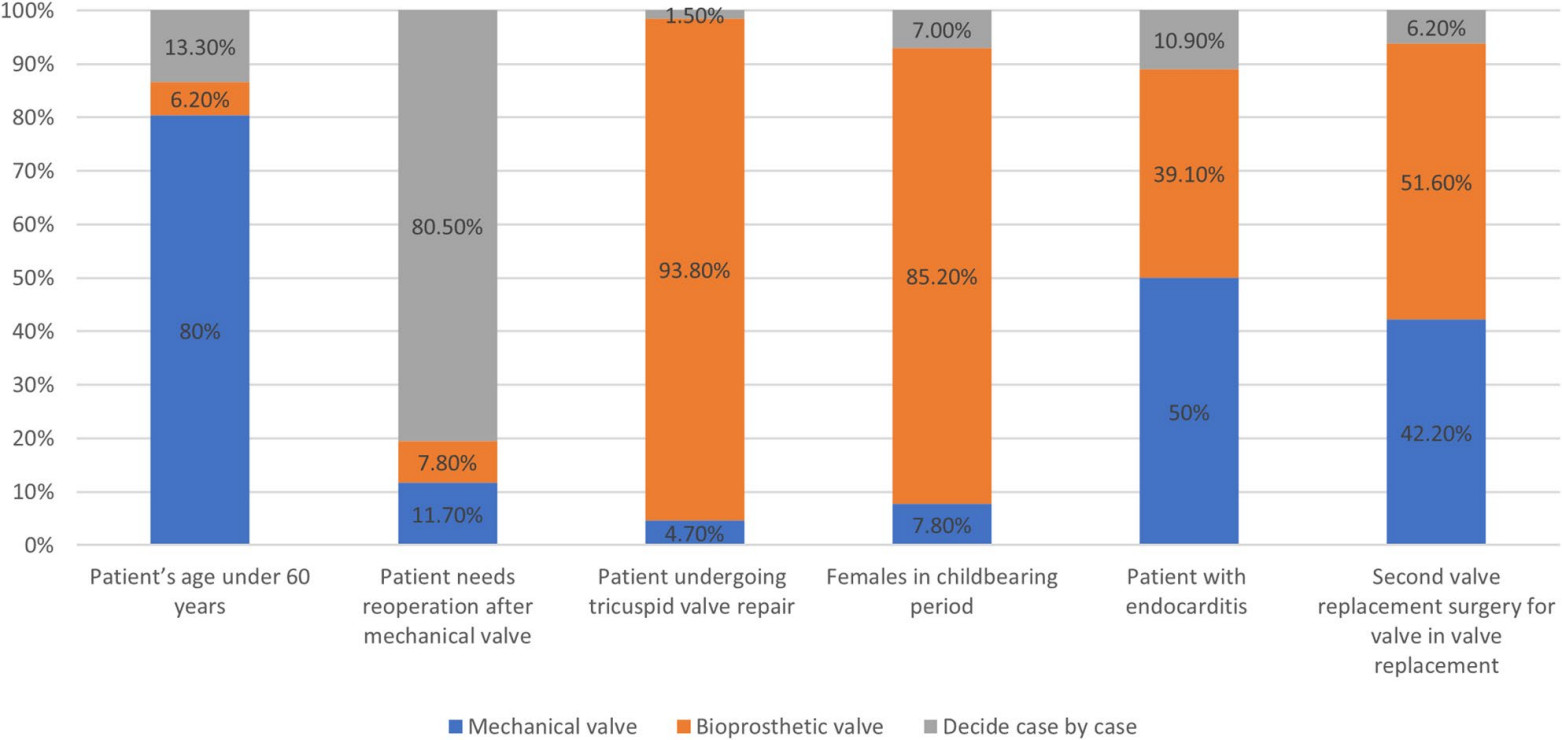
Preferred value types for various scenarios

Respondents were asked to indicate their preferred valve type in various clinical scenarios including patients under 60 years, reoperation after mechanical valve failure, tricuspid valve repair, females in childbearing age, patients with endocarditis, and valve in valve re-interventions. In patients under 60 years, there was a strong preference for using mechanical valves. In contrast, in reoperations after mechanical valve failure, decisions were more case-specific with no dominant preference. In tricuspid valve repair and females in childbearing period, most respondents preferred using biorposthetic valves. For patients with endocarditis and valve in valve surgeries, there was no clear preference for a specific valve type. Figure 2 presents a summary of expert preferences for valve types across various scenarios.

Respondents were asked to report which valve type is associated with a higher risk of some of the most common complications, including bleeding, stroke, thromboembolism, and endocarditis. The majority reported higher risks with mechanical valves, with 81% for bleeding, 77% for stroke, 84% for thromboembolism, 48% for endocarditis. However, some respondents indicated no significant difference in risk of complications between both types ranging from 13.3% in thromboembolism to 41% in endocarditis.

### Expert insights

In the expert insights section, respondents were asked to describe the drawbacks they encounter when using mechanical and bioprosthetic valves. For mechanical valves, the most frequently reported concerns were anticoagulation-related issues, thromboembolic complications, challenges with patient compliance to anticoagulation, and bleeding risks. While for bioprosthetic valves, the primary drawbacks were limited durability and structural degeneration, high cost, limited availability, risk of infection, and patient-related concerns, particularly fear of needing reoperation.

### Egypt versus global context

Respondents’ perceptions of the applicability of using global valve outcome data showed notable variability. While 39% believed that global data is somewhat representative and can be cautiously used, 25% said it is reflective of the case in Egypt, 23% stated that global data is not reflective of the population in Egypt due to social and cultural differences that may alter the outcomes, and 12.5% did not have an opinion on this.

## Discussion

Relevant findings emerged from both the narrative review and the expert survey on the pattern and outcomes of mechanical and bioprosthetic valves in Egypt. Following the completion of both components, outcomes were consolidated to formulate the final conclusions and develop actions for optimizing prosthetic valves utilization in Egypt.

The literature indicates that while outcome data for mechanical valves is relatively well-documented, there is a notable gap in evidence about bioprosthetic valve outcomes, especially reoperation rates, which is a critical factor in the valve selection decision. This is largely attributed to their lower utilization in Egypt, which was further confirmed by the expert survey findings.

While global data may be somewhat representative of valve outcomes in Egypt, notable differences are observed, so relying solely on it may be misleading. For instance, in contrast to global evidence, where bioprosthetic valves are associated with higher reoperation rates, Egyptian studies report higher reoperation rates with mechanical valves. However, these findings are based on only two studies in females of childbearing age and may not be generalizable.

Bleeding rates are consistently lower with bioprosthetic valves both globally and locally. Notably, absolute bleeding rates for mechanical valves appear lower in Egypt than in global reports. This observation has been suggested by the experts to be related to lower compliance with anticoagulation with mechanical valves in Egypt, a factor that might contribute to mechanical valves’ shorter longevity in Egypt as advised by the experts. However, this was not directly assessed in the present study.

Stroke and survival rates do not differ significantly between mechanical and bioprosthetic valves both in Egyptian studies and globally.

The expert survey showed that mechanical valves are the predominant choice among cardiothoracic surgeons in Egypt, partly due to their better accessibility, and partly due to their confidence in their superior durability- averaging more than 20 years, compared to approximately 9 years with bioprosthetic valves, according to their perceptions.

However, bioprosthetic valves are gaining traction among more experienced surgeons. A greater proportion of surgeons with over 20 years of experience preferred bioprosthetic valves compared to those with fewer years of practice.

There was consensus that multiple factors influence valve selection, with “patient age”, “childbearing potential”, and “ability to comply with anticoagulation” emerging as the most dominant. In Egypt, compliance to anticoagulation is not well documented in local studies, however experts estimated compliance to anticoagulation at around 80%. While this level of compliance is generally considered adequate, there is still a proportion of patients who are non-compliant, potentially leading to a shorter valve lifetime, and an increased risk of complications.

Surgeons indicated that they prefer mechanical valves for their superior longevity, especially for younger patients. However, they acknowledged their drawbacks, mainly related to anticoagulation, bleeding, and thromboembolic events. In certain cases, surgeons prefer bioprosthetic valves, such as elderly patients and females of childbearing age. Nonetheless, they emphasized that the decision is also affected by the shorter durability of these valves, higher relative cost, and limited availability in Egypt. They also indicated that, according to global evidence, novel bioprosthetic valves may represent a viable cost-effective option due to their improved durability and longer expected lifespan.[41] However, corresponding local evidence from Egypt remains limited. There was consensus that valve selection decision should be personalized weighing the advantages and disadvantages of each valve type for the patient.

The survey and the narrative review highlighted that there are persisitent gaps in areas such as patient compliance, affordability, and system-level challenges.

The experts recommended to develop an individualized decisionmaking tool to support valve selection. This tool should include all patient characteristics, not just age to help identify the most efficient valve for each patient. Similar tools have been developed already, however, the primary challenge lies in their implementation and validation of their outcomes to enable formal adoption by regulatory bodies.

Additionally, they emphasized the need for further research to generate local evidence-based data to inform clinical decisions. Finally, they recommended conducting cost-effectiveness studies across various patient profiles to asssess a holistic view about valve selection including complications, costs, reoperation rates, quality of life, and other related outcomes.

Our study came with some limitations, especially related to data scarcity and heterogeneity. Not all included studies reported outcomes for comparable patient populations. Consequently, while comparisons highlighted trends and differences, the ability to perform a quantitative synthesis was limited. Although some studies involved relatively large sample sizes, the overall number of studies reporting each outcome and the heterogeneity in terms of valve location, patient age, gender distribution, atrial fibrillation status, and other clinical characteristics restricted the feasibility of robust quantitative analyses. However, the reported values provide a meaningful overview of the available evidence in Egypt, and allow for broad, contextual comparisons.

Due to including data from multiple sources, outcome trends may not follow a consistent pattern over time. In some cases, data points for a specific outcome came from a single study, which might introduce bias due to study-specific conditions. Additionally, certain studies included patients undergoing reoperative (redo) surgeries, may have influenced the reported outcomes. Where redo procedures were explicitly reported, these values were highlighted, as they may be overestimated compared to outcomes in first-time surgery patients. Furthermore, some studies reporting outcomes for valve replacement populations may have included redo cases without clearly distinguishing them from primary procedures, potentially affecting the overall results. Additionally, most included studies did not report the follow-up completeness or number of patients at risk at the end of the study.

In the expert survey, respondents varied in the years of experience, clinical exposure, and location, which may introduce variability. However, this is also considered as a strength, as it makes the study more representative of the whole country not a specific hospital or surgeon. Additionally, the survey outcomes might have been affected by recall and confirmation bias due to self-reported data; however, the large sample size is expected to mitigate the impact of individual-level biases.

This study includes expert estimates for parameters such as valve durability and anticoagulation compliance, which were based on clinicians’ experience rather than directly measured patient-level data. As such, these findings are subject to recall and perception bias and should not be interpreted as precise clinical outcomes. However, these estimates were collected from a large and diverse group of 128 cardiothoracic surgeons practicing across multiple centers in Egypt and were intended to complement evidence from the literature. Therefore, they may provide useful contextual insights and approximate real-world expectations in the absence of robust local data, but should be interpreted with caution.

## Conclusion

Mechanical heart valves are predominantly used in Egypt for most patient profiles undergoing surgical valve replacement. Bioprosthetic valves may offer advantages for specific patient populations in Egypt. Relying solely on global outcome data of valve surgeries is not sufficient, as local outcomes may differ especially related to reoperation rates and patients’ behavior. There is a need for more local data on valve replacement outcomes. Ultimately, valve selection should be individualized, taking into account patient characteristics, behavior, cost, quality of life and all related factors.

## Supplementary Information

Supplementary Material 1

Supplementary Material 2

Supplementary Material 3

Supplementary Material 4

Supplementary Material 5

## Data Availability

All data generated or analysed during this study are included in this published article and its supplementary information files.

## Abbreviations

CI: Confidence Interval
INR: International Normalized Ratio
LMICs: Low- and Middle-Income Countries
OR: Odds Ratio
RCTs: Randomized Controlled Trials
SAVR: Surgical Aortic Valve Replacement
SMVR: Surgical Mitral Valve Replacement
TAVI: Transcatheter Aortic Valve Implantation
