# Supplementary material for: Outcomes of mechanical and bioprosthetic surgical valve replacements in egypt: evidence from a narrative review and experts’ survey
Source: J Cardiothorac Surg. 2026 Jun 10;21:573. doi: 10.1186/s13019-026-04400-6 (PMC13474610; doi:10.1186/s13019-026-04400-6)
Supplement: Supplementary file 1 [file 13019_2026_4400_MOESM1_ESM.docx]

Outcomes of Mechanical and Bioprosthetic Surgical Valve Replacements in Egypt: Evidence from a Literature Review and Experts’ Survey

Appendix 1: Population, Intervention, Comparator, Outcomes, and Study design (PICOS)

| Criterion | Inclusion criteria | Exclusion criteria |
| --- | --- | --- |
| Patients | -Adult patients (≥18 years) who underwent aortic or mitral valve replacement surgery | -Children (mean age in study <18)  -No valve replacement surgery  -Pulmonary or tricuspid valve replacement |
| Intervention | -Surgical valve replacement with mechanical valves or tissue valves | -Studies that do not include surgical valve replacement with mechanical or tissue valves (e.g., Transcatheter heart valves, TAVI/TAVR Valves, Homograft (Allograft) valves, autografts (Ross Procedure) or valve repair). |
| Comparator | -Not applicable | -Not applicable |
| Outcomes | -Reoperation rates  -Mortality or survival  -Stroke  -Bleeding events  -Thromboembolic events  - Compliance to anticoagulation | -Studies not differentiating outcomes by valve type  -Outcomes influenced by valve replacement and another factor (e.g., another drug/ haematological disease)  -No relevant outcomes |
| Study design | -Observational studies -Interventional studies  -Case series > 10 patients -Reports -Posters/ abstracts -Theses -Dissertations -registries/databases Systematic reviews and meta-analyses were included to check references for any missed references (snowballing), but no data were extracted directly from them | -Case reports -Case series with ≤10 patients -Non-systematic reviews -Protocols -Books -Surveys -Questionnaires -Model-based studies |
| Language | -Arabic or English studies | -Any other language |
| Region | -Egypt | -Any other country |
| Publication date | -Published after January 1, 2010 | -Published before January 1, 2010 |
