# Supplementary material for: Outcomes of mechanical and bioprosthetic surgical valve replacements in egypt: evidence from a narrative review and experts’ survey
Source: J Cardiothorac Surg. 2026 Jun 10;21:573. doi: 10.1186/s13019-026-04400-6 (PMC13474610; doi:10.1186/s13019-026-04400-6)
Supplement: Supplementary file 2 [file 13019_2026_4400_MOESM2_ESM.docx]

**Expert Questionnaire: Mechanical vs. Bioprosthetic Valve Outcomes in Egypt**

Dear Colleagues,

On behalf of the Egyptian Society of Cardiothoracic Surgery, you are kindly invited to participate in this questionnaire. This questionnaire is part of a study aimed at evaluating the real-world clinical outcomes of Surgical Aortic Valve Replacement (SAVR) and Surgical Mitral Valve Replacement (SMV) using mechanical versus bioprosthetic heart valves in Egypt. The goal is to validate findings from a recent targeted literature review and compare local expert opinions with global evidence. Your insights as an expert will help us better understand the applicability of international data to the Egyptian context, identify outcome gaps, and inform future decision-making.

The questionnaire consists of 25 structured questions, grouped into thematic sections: (1) Demographics, (2) Valve selection criteria and preferences, (3) Clinical outcomes and complications (e.g., reoperation, bleeding, stroke), (4) Experts insights, and (5) Comparisons between Egyptian and global outcomes. Most questions are multiple-choice. A few questions require short, one- or two-word answers to capture key expert reflections.

Please respond based on your clinical experience and insights from your practice. Your participation is essential to building an accurate and evidence-informed understanding of valve replacement outcomes in Egypt.

**☐ *By submitting this questionnaire, you confirm your consent to use your anonymized responses for research purposes.***

## Section1: Demographics

1. What is your primary practice setting or affiliation?
   - University/Academic hospital
   - Government/Public hospital
   - Private hospital/clinic
   - Other (please specify)
2. How many years have you been a practicing physician?
   - < 5 years
   - 5–10 years
   - 11–20 years
   - 20 years +
3. What is your current professional title?
   - Resident
   - Specialist
   - Consultant

## Section 2: Valve Selection Criteria and Preferences

4. In your practice, which type of valve do you **use more frequently** for valve replacements?
   - Mostly **mechanical** valves
   - Mostly **bioprosthetic (tissue)** valves
   - No strong preference (use both about equally)
5. Over the past 5–10 years, how has your use of bioprosthetic valves **changed**?
   - **Increased** (using bioprosthetics more often now)
   - **Decreased** (using bioprosthetics less often now)
   - **No significant change** in usage
6. When selecting a mechanical valve, what are the **most important factor/s** influencing your choice about valve type? (Multiple answers allowed)

☐Patient **age** (younger vs. older)

☐Gender

☐ Being a female in a childbearing age

☐Anticoagulation ability/compliance (risk of non-compliance)

☐ Patient **comorbidities** / life expectancy

☐ Valve **cost** or patient affordability

☐ Availability of a specific type of valves

☐ Patient opinion

☐ Patient **preference**, lifestyle considerations or Occupation

☐ Patient’s socioeconomic status

☐ Having an active/ sedentary lifestyle

☐ Atrial fibrillation

☐ Warfarin toxicity

☐ Warfarin resistance

☐ Valve size

☐ Other (please specify)

7. When selecting a bioprosthetic valve, what are the **most important factor/s** influencing your choice about valve type? (Multiple answers allowed)

☐Patient age (younger vs. older)

☐Gender

☐ Being a female in a childbearing age

☐Anticoagulation ability/compliance (risk of non-compliance)

☐ Patient comorbidities / life expectancy

☐ Valve cost or patient affordability

☐ Availability of a specific type of valves

☐ Patient opinion

☐ Patient preference or lifestyle considerations

☐ Patient’s socioeconomic status

☐ Having an active/ sedentary lifestyle

☐ Atrial fibrillation

☐ Warfarin toxicity

☐ Warfarin resistance

☐ Valve size

☐Being a TAVI friendly valve

☐ Other (please specify)

8. At approximately what **patient age** do you consider **a bioprosthetic valve** over a mechanical valve for your patient?
   - **20-40** years
   - **40**-**50** years
   - **50–60** years
   - **60–70** years
   - **70+** years
   - No fixed age (depends on individual factors)

## Section 3: Clinical Outcomes and Complications

9. **From real life practice in Egypt,** approximately how many **years** does a bioprosthetic valve typically last in a middle-aged patient before needing replacement (reoperation)?
   - **………..**
10. **From real life practice in Egypt,** approximately how many **years** does a mechanical valve typically last in a middle-aged patient before needing replacement (reoperation)?
    - **………..**
11. For patients under 60, which valve type do you believe offers **better long-term survival** (e.g. 5–10 year survival)?
    - Mechanical valves
    - Bioprosthetic valves
    - No significant difference in long-term survival
12. If a patient’s initial surgery was with a **mechanical valve** and it **fails/needs reoperation** (e.g. due to thrombosis or other complication), what is your choice for the replacement valve in the second surgery?
    - Replace with another **mechanical** valve
    - Switch to a **bioprosthetic** valve
    - Decide case-by-case (depends on circumstances)
13. What proportion of your patients with mechanical valves are **compliant** with anticoagulation (e.g. Warfarin)?
    - **………. %** of patients
14. In your experience, which valve type is associated with a higher risk of **bleeding** complications?
    - Mechanical valves
    - Bioprosthetic valves
    - No major difference in bleeding risk
15. In your experience, which valve type is associated with a higher risk of **stroke** (thromboembolic stroke)?
    - Mechanical valves
    - Bioprosthetic valves
    - No significant difference in stroke risk
16. In your experience, which valve type has a higher risk of **other thromboembolic events** (e.g. valve thrombosis or systemic emboli, aside from stroke)?
    - Mechanical valves
    - Bioprosthetic valves
    - No significant difference in thromboembolic risk
17. In your experience, which type of valve has a higher incidence of **prosthetic valve endocarditis** (infection of the valve)?
    - Mechanical valves
    - Bioprosthetic valves
    - No significant difference in endocarditis risk
18. *If the patient has a tricuspid valve disease needing replacement, what type of valve do you prefer?*
    - Mechanical valves
    - Bioprosthetic valves
    - Other details, please specify
19. *In females in the childbearing period, what type of valve do you prefer?*
    - Mechanical valves
    - Bioprosthetic valves
    - Other details, please specify
20. *If the patient has endocarditis, what type of valve do you prefer?*
    - Mechanical valves
    - Bioprosthetic valves
    - Other details, please specify
21. *Which is your preferred prosthesis for the second valve replacement surgery when doing valve in valve procedure?*
    - Mechanical valves
    - Bioprosthetic valves
    - Other details, please specify

## Section 4: Expert Insights

22. What do you consider the biggest **drawbacks of mechanical valves** in Egypt?

………………..

23. What do you consider the biggest **drawbacks of bioprosthetic valves** in Egypt?

*……………….*

## Section 5: Egypt vs. Global Context

24. Do you feel that outcomes reported in **international literature** (for mechanical vs. bioprosthetic valves) **reflect real-world outcomes** in Egypt?
    - **Yes** – global data is generally reflective of outcomes in Egypt
    - **Somewhat** – partially reflective, but with notable differences
    - **No** – outcomes in Egypt differ significantly from global data
    - Not sure / no opinion
25. If you perceive differences in outcomes for Egypt, what are the key **factors** you believe are responsible? *(one or two words, e.g. “compliance”)*

*………………..*
