# Supplementary material for: Outcomes of mechanical and bioprosthetic surgical valve replacements in egypt: evidence from a narrative review and experts’ survey
Source: J Cardiothorac Surg. 2026 Jun 10;21:573. doi: 10.1186/s13019-026-04400-6 (PMC13474610; doi:10.1186/s13019-026-04400-6)
Supplement: Supplementary file 3 [file 13019_2026_4400_MOESM3_ESM.docx]

Outcomes of Mechanical and Bioprosthetic Surgical Valve Replacements in Egypt: Evidence from a Literature Review and Experts’ Survey

Appendix 4: Odds ratios of outcomes of interest reported by Singab at al.

| Outcome | Odds ratio (Bioprosthetic valves vs mechanical valves) | Confidence interval |
| --- | --- | --- |
| Re-exploration for bleeding | **0.21*** | 0.1047–0.4023 |
| Long-term mortality (10-years) | **0.32*** | 0.1252–0.8352 |
| Reoperation | 0.14 | 0.0175–1.1802 |
| Stroke | 0.77 | 0.2624–2.2733 |
| Short-term Mortality (30-days) | 0.52 | 0.0465–5.7576 |

*Significant effect at 95% confidence interval
