# Supplementary material for: Outcomes of mechanical and bioprosthetic surgical valve replacements in egypt: evidence from a narrative review and experts’ survey
Source: J Cardiothorac Surg. 2026 Jun 10;21:573. doi: 10.1186/s13019-026-04400-6 (PMC13474610; doi:10.1186/s13019-026-04400-6)
Supplement: Supplementary file 4 [file 13019_2026_4400_MOESM4_ESM.docx]

Outcomes of Mechanical and Bioprosthetic Surgical Valve Replacements in Egypt: Evidence from a Literature Review and Experts’ Survey

Appendix 2: Data extraction sheet components

| Domain | Contents |
| --- | --- |
| General study characteristics | - Title - Author_year - Final decision - Reason for exclusion - Source of search - Link - Type of study - Total number of patients with surgery - Country/ countries |
| Subgroup data | - Subgroup name - Number of patients in subgroup - Single arm or comparative outcome |
| Single arm outcomes | - Outcome category - Name of outcome - Timepoint of outcome (From operation) - Timepoint unit - Type of valve - Risk of bleeding - Type of numerical age value - Average age - Age range - Type of age dispersion measure - Numerical value of dispersion - Male % - 1st operation type of implant - 2nd operation (reoperation) type of implant - Adjustment for confounding (observational) OR Randomization (interventional) - Type of numerical value - Numerical value of outcome - Type of dispersion measure - Numerical value of dispersion - Qualitative description of outcomes |
| Comparative outcomes | - Type of comparative outcome - Reference group - Intervention group - Adjustment for confounding (observational) OR Randomization (interventional) - Numerical value of comparative outcome - Type of dispersion measure - Numerical value of dispersion |
| Comments | - Any additional comments on study characteristics, population or outcomes |
