# Supplementary material for: Outcomes of mechanical and bioprosthetic surgical valve replacements in egypt: evidence from a narrative review and experts’ survey
Source: J Cardiothorac Surg. 2026 Jun 10;21:573. doi: 10.1186/s13019-026-04400-6 (PMC13474610; doi:10.1186/s13019-026-04400-6)
Supplement: Supplementary file 5 [file 13019_2026_4400_MOESM5_ESM.docx]

Outcomes of Mechanical and Bioprosthetic Surgical Valve Replacements in Egypt: Evidence from a Literature Review and Experts’ Survey

Appendix 5: Expert survey responses

# Section 1: Experts’ demographics (Q1-Q3)

# Section 2: Valve Selection Criteria and Preferences (Q4-Q8)

# Section 3: Clinical Outcomes and Complications (Q9-Q21)

9. **From real life practice in Egypt,** approximately how many **years** does a bioprosthetic valve typically last in a middle-aged patient before needing replacement (reoperation)?

10. **From real life practice in Egypt,** approximately how many **years** does a mechanical valve typically last in a middle-aged patient before needing replacement (reoperation)?

Mechanical valves demonstrate significantly longer durability, lasting a mean of 20.8 ± 8.2 years, compared to bioprosthetic valves, which last a mean of 9.0 ± 2.9 years.

13. What proportion of your patients with mechanical valves are **compliant** with anticoagulation (e.g. Warfarin)?

**72.7% ± 26.2%** of patients with mechanical valves were compliant with anticoagulation therapy.

# Section 4: Expert Insights (Q22-Q23)

22.What do you consider the biggest **drawbacks of mechanical valves** in Egypt?

| Theme | n | % | Examples of drawbacks mentioned |
| --- | --- | --- | --- |
| Anticoagulation-related issues | 64 | 50.0 | Lifelong anticoagulation needs, INR monitoring difficulties, warfarin toxicity, bleeding risk, socioeconomic barriers |
| Thrombosis & thromboembolic complications | 24 | 18.8 | Valve thrombosis, stuck valve, thromboembolic events, pannus formation |
| Compliance & patient-related issues | 20 | 15.6 | Poor compliance, low literacy, lack of follow-up, young women concern |
| Bleeding complications | 12 | 9.4 | General bleeding, bleeding with noncompliance, mitral bleeding |
| Availability & cost issues | 5 | 3.9 | Limited availability, high cost, price fluctuations |
| Other issues | 3 | 2.3 | Valve noise, prosthetic dysfunction, limited options |

23. What do you consider the biggest **drawbacks of bioprosthetic valves** in Egypt?  ?

| Theme | N | % | Examples of drawbacks mentioned |
| --- | --- | --- | --- |
| Limited durability and structural degeneration | 70 | 54.7 | Early degeneration, SVD, short life span, frequent reoperation, dysfunction |
| High cost and economic issues | 30 | 23.4 | High price, financial burden, affordability challenges |
| Availability and accessibility issues | 20 | 15.6 | Limited availability, poor storage, lack of types |
| Risk of infection and complications | 5 | 3.9 | Endocarditis, thrombosis, pannus |
| Patient-related issues and fear of redo surgery | 3 | 2.3 | Patient fear of reoperation, poor follow-up |
| Other / not specific | 2 | 1.6 | "Nothing", "No specific", general comments |

# Section 5: Egypt vs. Global Context (Q24-Q25)

25. If you perceive differences in outcomes for Egypt, what are the key **factors** you believe are responsible? *(one or two words, e.g. “compliance”)*

| Theme | n | % | Examples of factors mentioned by physicians |
| --- | --- | --- | --- |
| Compliance & patient-related | 55 | 43.0 | Compliance, follow-up, lifestyle, cultural levels, patient knowledge |
| Socioeconomics & cost | 11 | 8.6 | Cost, affordability, socioeconomic standards, financial issues |
| Anticoagulation-related | 10 | 7.8 | Anticoagulation control, warfarin monitoring, INR follow-up |
| Health system & data | 9 | 7.0 | Availability of valves, lack of registries, poor follow-up systems |
| Pathology-related | 6 | 4.7 | Rheumatic fever, different pathology, age-related factors |
| Education & awareness alone | 5 | 3.9 | Patient awareness, lack of education, less education |
| Valve type & durability | 5 | 3.9 | Type of bio prosthesis, mechanical valve, early degeneration |
| No difference / no opinion | 16 | 12.5 | "No difference", "no opinion", "same outcome" |
| Other | 11 | 8.6 | Adjustable research, surgeon experience, data bias, miscellaneous |
